# In Vitro Screening of Antimicrobial and Anti-Coagulant Activities, ADME Profiling, and Molecular Docking Study of *Citrus limon* L. and *Citrus paradisi* L. Cold-Pressed Volatile Oils

**DOI:** 10.3390/ph16121669

**Published:** 2023-11-30

**Authors:** Assia Hamdi, Mabrouk Horchani, Hichem Ben Jannet, Mejdi Snoussi, Emira Noumi, Nouha Bouali, Adel Kadri, Flavio Polito, Vincenzo De Feo, Hayet Edziri

**Affiliations:** 1 Laboratory of Chemical, Pharmaceutical and Pharmacological Development of Drugs, Faculty of Pharmacy, University of Monastir, Monastir 5000, Tunisia; hamdiessia@gmail.com; 2 Laboratory of Heterocyclic Chemistry, Natural Products and Reactivity, Medicinal Chemistry and Natural Products (LR11ES39), Department of Chemistry, Faculty of Science of Monastir, University of Monastir, Avenue of Environment, Monastir 5019, Tunisia; horchani.mabrouk@gmail.com (M.H.); hichem.bjannet@gmail.com (H.B.J.); 3 Department of Biology, College of Science, University of Ha’il, Hail 2440, Saudi Arabia; eb.noumi@uoh.edu.sa (E.N.); nouhabouali82@gmail.com (N.B.); 4 Medical and Diagnostic Research Centre, University of Ha’il, Hail 55473, Saudi Arabia; 5 College of Science and Arts in Baljurashi, Al Baha University, Al Baha 65527, Saudi Arabia; lukadel@yahoo.fr; 6 Laboratory of Plant Biotechnology Applied to Crop Improvement, Faculty of Sciences of Sfax, University of Sfax, Sfax 3000, Tunisia; 7 Department of Pharmacy, University of Salerno, Via Giovanni Paolo II, 132, 84084 Fisciano, Italy; fpolito@unisa.it; 8 Laboratory of Transmissible Diseases and Biologically Active Substances, Faculty of Pharmacy, Monastir 5000, Tunisia; jaziri_hayet@yahoo.fr

**Keywords:** *Citrus* species, essential oils, cold press, antibacterial, anti-coagulant, docking study, ADME

## Abstract

*Citrus*, which belongs to the Rutaceae family, is a very widespread genus in the Mediterranean Basin. In Tunisia, various parts of these spontaneous or cultivated plants are used in common dishes or in traditional medicine. The purpose of this work was to investigate *C. limon* and *C. paradisi* essential oil (EO). The samples were studied for their chemical composition using SPME/MS, as well as their antibacterial and antifungal activities. Prothrombin time (PT) and activated partial thromboplastin time (aPTT) methods were used to evaluate the anticoagulant potentialities. The obtained results show that both essential oils are rich in monoterpenes hydrocarbons, whereby limonene is the main compound in *C. paradisi* EO (86.8%) and *C. limon* EO (60.6%). Moreover, *C. paradisi* EO contains *β*-pinene (13.3%), sabinene (2.2%) and *α*-pinene (2.1%). The antibacterial assay of the essential oils showed important bactericidal and fungicidal effects against all strains tested. In fact, the MICs values of *C. limon* EO ranged from 0.625 to 2.5 mg/mL against all Gram-positive and Gram-negative bacteria, and from 6.25 to 12.5 mg/mL for *Candida* spp. strains, while *C. paradisi* EO was more active against all bacteria with low MICs values ranging from 0.192 to 0.786 mg/mL, and about 1.5 mg/mL against *Candida* species. Both tested *Citrus* EOs exhibited interesting anticoagulant activities as compared to heparin. The molecular docking approach was used to study the binding affinity and molecular interactions of all identified compounds with active sites of cytidine deaminase from *Klebsiella pneumoniae* (PDB: 6K63) and the C (30) carotenoid dehydrosqualene synthase from *Staphylococcus aureus* (PDB: 2ZCQ). The obtained results show that limonene had the highest binding score of −4.6 kcal.mol^−1^ with 6K63 enzyme, and −6.7 kcal.mol^−1^ with 2ZCQ receptor. The ADME profiling of the major constituents confirmed their important pharmacokinetic and drug-like properties. Hence, the obtained results highlight the potential use of both *C. limon* and *C. paradisi* essential oils as sources of bioactive compounds with antibacterial, antifungal, and anti-coagulant activities.

## 1. Introduction

Many serious health problems, such as heart and cardiovascular diseases, the emergence of nosocomial infections, and multidrug resistance in microorganisms, impact daily human life. Plant-derived molecules are promising alternatives used to counteract the effects of such human diseases [1,2]. As coagulation and thrombosis are among the major causes of atherosclerosis and cardiovascular diseases, numerous methods of treating or preventing thrombosis have been explored [3]. It is well documented that blood coagulation is directly associated with cardiovascular diseases via many factors, such as platelet activation and factor VIII level. Several commercial anticoagulant drugs have shown limited applicability because of their narrow therapeutic range. Their application doses can be considered unsafe as a result of their potential interactions with other drugs. Thus, due to interference with different medicines and certain negative effects, efforts are still being made to find new safe candidates for use as anticoagulants [3,4].

Plant essential oils are among the main sources of biological molecules considered promising, such as anticoagulant and antimicrobial agents. Different factors are implicated in the complexity of the chemical composition of citrus essential oils, such as genotype–cultivar [5], harvest year [6], harvest date, maturity of the species [7] and the extraction system [8]. This variability leads, in turn, to the different biological effects correlated with the major and minor compounds presented in these volatile oils. These studies have revealed that the fruits of three cultivar citrus species showed differences in terms of their biometrics and physico-chemical characteristics. The flavonoid contents also varied from one cultivar species to another. A study has demonstrated that these chemical compounds were similar but varied between areas [5]. Moreover, a great variability in the essential oil composition of *Citrus bergamia*, Risso was registered over thirty-five years, and this can be explained by several factor, such as the production year, the plant age, the microclimate, the cultivar, the techniques of agronomy, the harvest date of fruits, and the conditions and duration of storage [6]. Previous work has shown that cold-pressing, hydrodistillation and microwave-accelerated distillation can be used to extract essential oils, and these affect citrus fruits’ fragrance, yield and chemical composition [8]. 

Essential oil compounds can directly influence hemostasis or modify the antithrombotic action. They have antiplatelet and fibrinolytic or thrombin-inhibiting effects, induced by modifying prostaglandin levels, inhibiting cyclo-oxygenase, inhibiting platelet activating factor, etc. [4]. Volatile oil components are also known for their strong antimicrobial activity against bacteria, yeasts, and viruses. 

Citrus is among the most famous fruits, cultivated today in all Mediterranean regions. Fresh or dried, Citrus compounds are used in foods, flavors, refreshing drinks, pastries, and confectionery [9]. They are known as a potential source of bioactive natural molecules, such as phenolic compounds, minerals, citric acid, ascorbic acid, and terpenoids. Several works have shown their possible uses as antioxidant, anti-inflammatory, antipathogenic and anticancer substances [10]. They are also used as natural preservatives that preserve food from different oxidative or microbial alterations [11]. Some citrus extracts, such as aqueous extracts, essential oils and juice, have already been studied for their anticoagulant activity [3,12]. They are endowed with important broad-spectrum activities [13], and have revealed great potential in their use against bacteria and fungi [14,15]. *C. medica* (citron), *C. limon* (lemon), *C. reticulata* (mandarin, tangerine), *C. aurantium* (sour orange), *C. sinensis* (sweet orange), *C. clementina* (clementine) and *C. paradise* (grapefruit) are some of the citrus species cultivated around the world [16]. 

Tunisia is a country that produces cultivated or adapted citrus species. In 2019, the total production level of these plants in Tunisia was 393.3 thousand tones, divided between 135.0 thousand tons of oranges, 94.7 thousand tons of tangerines, 54.3 thousand tons of lemons and limes, and 109.3 thousand tons of grapefruits [17]. Different organs of these species are already widely used in traditional medicine, as well as for culinary and in economic purposes. The literature shows wide investigations into Tunisian citrus species [18,19]. 

New works have focused on in vitro experiments via the biological activity approach coupled to the computational approach, which combines molecular docking and ADMET prediction to estimate the biological importance of certain compounds present in essential oil [20,21,22]. Thus, the goal of the present research is to study the chemical composition, as well as the antibacterial and antifungal potentialities, of essential oils of *C. paradisi* L. and *C. limon* L. against multidrug-resistant bacteria and yeasts, and to determine their anticoagulant activities. In addition to the ADME analysis, a molecular docking investigation was undertaken to estimate the molecular interactions and binding affinities of all identified compounds with the target enzymes involved in antibacterial activities. 

## 2. Results

### 2.1. Chemical Composition of Essential Oils

The characterization of volatile oils was carried out by GC-SPME. Twenty-two and twenty components were identified in C. limon and C. paradise, representing 99.9% and 98.9% of the total identified compounds, respectively (Table 1). These compositions show that C. limon volatile oil was rich in monoterpene hydrocarbons (93.3%), followed by oxygenated monoterpenes (4.8%). In addition, C. paradisi EO was dominated by limonene (60.6%), β-pinene (13.3%) and γ-terpinene (11%). However, limonene, with a prevalence of 86.8%, was the main compound in C. paradisi EO. The obtained chromatograms are listed in Supplementary Material SM1.

### 2.2. Antibacterial Activity

The obtained results are summarized in Table 2. *C. limon* and *C. paradisi* EOs were found to be active against all tested microorganisms to different degrees. Interestingly, *C. limon* seems to have been more active against Gram-negative bacteria as compared to *C. paradisi* essential oil. The obtained minimal inhibitory concentrations (MICs) ranged between 3.84 and 12.5 mg/mL for both essential oils. 

Moreover, the two tested essential oils derived from *C. limon* and *C. paradisi* exhibited bactericidal effects against imipenem-resistant *Acinetobacter baumannii*, *Klebsiella pneumoniae*, *Pseudomonas aeruginosa* and *Escherichia coli*. Similarly, the minimal bactericidal concentration values (MBCs) varied between 1.25 and 2.5 mg/mL for *C. limon* EO, and between 0.192 and 1.536 mg/mL for *C. paradisi* EO. 

Our results also show that *C. limon* was active against Gram-positive bacteria such as methicillin-resistant *S. aureus* strains (MRSA-3 and MRSA-126), as well as *Enterococcus faecalis* ATCC 29212 and *E. faecium* (CI234). These MIC values were lower than those of the essential oil of *C. limon*, and ranged between 0.192 and 0.786 mg/mL. The MBC/MIC ratio was less than 4; therefore, the essential oils studied showed bactericidal effects against the bacterial strains tested [23].

### 2.3. Antifungal Activity

The results of the anticandidal activity are summarized in Table 3. *C. paradisi* EO exhibited significant antifungal activities against *C. albicans*, *C. krusei*, *C. parapsilosis* and *C. glabrata*, with the lowest MIC values (1.2 mg/mL), while the same *Candida* species were killed when using 2.4 mg/mL of *C. paradisi* EO.

Furthermore, the essential oil of *C. limon* showed moderate anticandidal activities, with MIC values varying from 6.25 to 12.5 mg/mL, and MFC values of about 12.5 mg/mL against all tested *Candida* species. The tested essential oils exhibited fungicidal activities against all tested yeast strains (MFC/MIC value < 4) [24].

### 2.4. Determination of Anticoagulant Activity

The anticoagulant activities of both essential oils were calculated using prothrombin time (PT) and activated partial thromboplastin time (aPTT) estimations. The results are summarized in Table 4. In fact, our results show that *C. limon* and *C. paradisi* EOs possessed the same prothrombin prolongation times, estimated at 80.00 ± 1.67 s and 82.21 ± 2.65 s, respectively.

However, *C. paradisi* EO showed the highest aPTT of about 180.00 ± 1.20 s. This value is higher than the aPTT obtained with heparin (120.50 ± 1.18 s), which was the positive control.

### 2.5. Molecular Docking Studies

*Klebsiella pneumoniae* is a Gram-negative nosocomial pathogen responsible for various types of healthcare-associated infections, such as urinary tract infections, sepsis, and bacteremia [25]. On the other hand, *S. aureus* (Gram-positive bacterium) is an opportunistic pathogen responsible for nosocomial infections and human disorders [26].

In light of the above facts and based on previous works, we can infer the importance of docking studies for determining antibacterial activity [27,28]. Molecular docking studies have been performed to explore the interactions via binding between the components of the samples biologically tested for their antibacterial potency and the active sites of the cytidine deaminase from *K. pneumoniae* (PDB ID: 6K63) on one side and *S. aureus* dehydrosqualene synthase (PDB ID: 2ZCQ) on the other side.

The results (Table 5) are laid out as the docking score (kcal/mol) of a ligand onto a target enzyme next to the interaction mechanisms (Van der Waals, H-bond, and hydrophobic interactions). Interestingly, a high degree of negativity in the binding affinity values suggests greater effectivity, and that the tested compound could be used in controlling the tested bacteria.

Table 6 suggests the ligands and receptors bind with interaction residues. Overall, in the context of the cytidine deaminase from *K. pneumoniae*, most of the compounds showed significant results, especially limonene (having the lowest free binding energy value, −4.6 kcal/mol).

**Figure 1:**
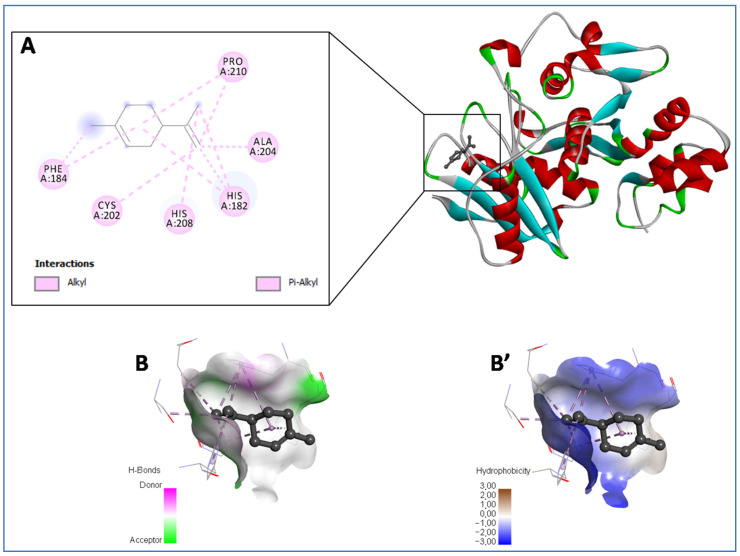
Representation of the best docked compounds bound to the pocket region of cytidine deaminase from *Klebsiella pneumoniae* (PDB ID: 6K63). Micrographs of the pocket region with hydrogen bond (**B**) and hydrophobicity illustrations (**B′**), and a corresponding 2D diagram of interactions (**A**).

Since the chain skeleton forming this molecule is devoid of heteroatoms, the formation of hydrogen bonds is impossible (Figure 1A,B,B’). The inhibition force of the target enzyme is perceptible through the strength of hydrophobic interactions (alkyl and Pi–alkyl interactions) formed with the residues His182, Phe184, Cys202, Ala204, His208 and Pro210. This significance is proven by the binding energy values of limonene, α-pinene and γ-terpinene, which exceed those of the standard docked ligand (ciproflo–acin), as Table 5 displays. However, α-pinene was found to be the second most active compound, with −4.4 kcal/mol, showing two alkyl interactions with Pro210, in addition to five Pi–alkyl interactions with His182 and Phe184 and Pi–Sigma with His182.

On the other hand, as the tabulated data show, γ-terpinene (−4.3 kcal/mol) is considered the third most active compound, exhibiting five alkyl interactions with residues Cys202, Ala204, Pro210 and Lys265, in addition to three Pi–alkyl interactions with His182 and Phe184 and a Pi–Sigma integration with His182. Sabinene, *β*-pinene and geranial compounds with binding energy values of −4.1 kcal/mol were found to be moderately active molecules compared to other compounds.

**Figure 2:**
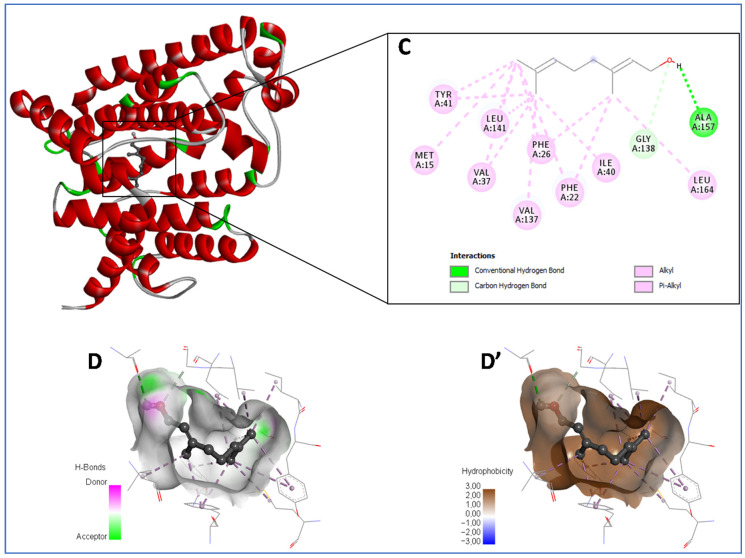
A representation of the best-docked compounds bound to the pocket region of *Staphylococcus aureus* dehydrosqualene synthase (PDB ID: 2ZCQ). Micrographs of the pocket region with hydrogen bond (**D**) and hydrophobicity illustrations (**D′**), and the corresponding 2D diagram of interactions (**C**).

Regarding *S. aureus*’s dehydrosqualene synthase–ligand complex, the obtained results show that geranial at −6.9 kcal/mol was the most potent compound, followed by γ-terpinene (−6.8 kcal/mol) then limonene (−6.7 kcal/mol), compared to other docked ligands in the standard (Ciprofloxacin). As shown in Figure 2C,D,D’, geranial is involved in a conventional hydrogen bond with Ala157 through its hydroxyl group, in addition to some hydrophobic interactions (alkyl and Pi–alkyl) with a sequence of amino acids—Met15, Phe22, Phe26, Val37, Ile40, Tyr41, Val137, Leu141 and Leu164—in addition to a carbon–hydrogen bond with Gly138. Further, the potential enzyme-inhibiting action of γ-terpinene is perceivable through its several hydrophobic interactions with more or less the same residues as geranial, while α-pinene (−5.7 kcal/mol) was found to be the least active compound, although it engaged in multiple hydrophobic interactions as detailed in Table 5.

### 2.6. Predictive ADME Analysis

The ADME (absorption, distribution, metabolism, and excretion) properties of the six predominant constituents of the tested *Citrus* EOs were studied, including their pharmacokinetic and drug-like properties, using Swiss ADME online server (http://www.swissadme.ch/, accessed on 7 August 2023). Indeed, the tested compounds (Table 7) were found to adhere to Lipinski’s rule, and also share topological polar surface area (TPSA) values below 30 Å2, thus exhibiting good brain penetration in addition to good lipophilicity behavior, which is expressed by their consensus Log Po/w in the range 2.74–3.44. Their bioavailability score of 0.55 displays their greater drug-like properties.

**Figure 3:**
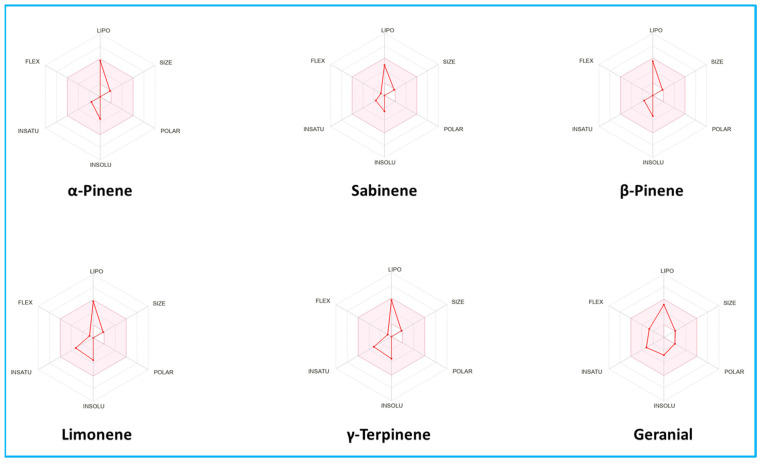
Bioavailability radar of the selected phytoconstituants.

**Figure 4:**
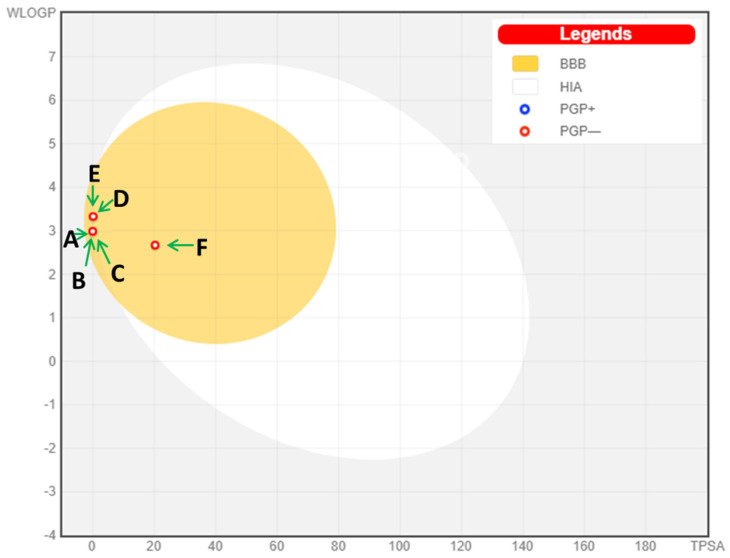
Boiled-egg graph of the selected phytoconstituants: A—α-Pinene, B—Sabinene, C—β-Pinene, D—Limonene, E—γ-Terpinene and F—Geranial.

As shown in the tabulated data, all compounds achieved low gastrointestinal absorption (GI) and easily passed the blood–brain barrier (BBB), meaning they could bind to specific receptors. Furthermore, the study’s components interacted the most with isoenzymes of the Cytochrome P (CYP) family, demonstrating their greater effectiveness and insignificant toxicity. Moreover, in their radar plots (Figure 3), all tested compounds were entirely within the pink zone, confirming their greater drug-likeness along with a good bioavailability profile. Besides this, in the boiled egg graph (Figure 4), the compounds A (α-pinene), B (sabinene), C (β-pinene), D (limonene), E (γ-terpinene) and F (geranial) all appear in the yellow region (yolk), with a red point confirming their high capacity for brain penetration, with “BB” acting as a non-substrate of P–gp.

## 3. Discussion

In this work, we have studied the chemical compositions and antimicrobial and anticoagulant activities of two *Cirus* plant species: *C. limon* and *C. paradisi*. Our results reveal the identification of two different chemotypes: *C. limon* (limonene/β-pinene chemotype; 606/13.3%) and *C. paradisi* (Limonene 86.6%). A literature survey has indicated that the volatile oil of *C. limon* collected from the Cap Bon, Tunisia contained limonene (39.74%) and β-pinene (25.44%) [11], while *C. limon* EO from Menzel Bouzelfa (Northeast Tunisia) was rich in limonene (37.63–69.71%), β-pinene (0.63–31.49%), γ-terpinene (0.04–9.96%), and *p*-cymene (0.23–9.84%) [29]. Previous studies on cultivated species *C. limon* collected from northeastern India indicated that the major compounds were limonene (55.40%) and neral (10.39%), followed by trans-verbenol (6.43%) and decanal (3.25%) [30]. Limonene (52.77%), geranyl acetate (9.92%), and trans-limonene oxide (7.13%) were found to be the major components of *C. limon* EO from Picos City, state of Piaui, Brazil [31].

Using Gas Chromatography–Mass Spectrometry, essential oils of *C. limon* and *C. paradisi* purchased from Ravetllat Aromatics (Barcelona, Spain) showed limonene (69.9%), followed by β-pinene (11.2%) and γ-terpinene (8.21%), and limonene (96.2%) and myrcene (1.5%), as their main compounds, respectively [32].

Moreover, *C. paradisi* EO from Kukeng, Yunlin County, southern Taiwan, obtained by hydro-distillation and cold-pressing, showed limonene at contents of 96.06% and 92.83%, respectively [33]. D-limonene (75.05%) and β-myrcene (7.25%) were the major components of *C. paradisi* EO purchased from Onilegogoro market, Mushin, Lagos, West Nigeria [34,35]. In addition, *C paradisi* EO obtained via cold-pressing extraction by the company Frutech International Corporation de México S.A. showed limonene as its major compound (area = 94.4%) [36].

Overall, the results of previous studies on *C. limon* and *C. paradisi* EOs from various countries and regions show that, in most cases, limonene (monoterpene hydrocarbon) was the major compound, which results agree with our findings on the characteristics of citrus. A small variability has been recorded in the compositions of minor components. This variability can be explained by the differences in environmental conditions, such as temperature, rainfall and soil composition.

The investigation of the antibacterial effects against a wide range of bacteria showed that *C. paradisi* EO was more active than *C. limon* EO. This important antibacterial activity of EOP may be explained by its richness in limonene (86.8%) and oxygenated monoterpenes (9.7%) compared to EOL, comprising 60.6% limonene and 4.8% oxygenated monoterpenes.

Concerning the antibacterial activities of the obtained citrus EOs, our results show that *C. limon* and *C. paradisi* EOs were active against Gram-positive and Gram-negative bacteria to different degrees. Previous reports on *C. limon* EO show that it was active against Gram-positive bacteria, with MIC and MBC values against *S. aureus* of about 0.023 mg/L and 0.011 mg/L, respectively [37]. Using both disc diffusion (inhibition zone determination—IZ) and microdilution (MIC and MBC value determination) assays, Ben Hsouna and colleagues [11] reported that *C. limon* EO was active against *Bacillus subtilis* ATCC 6633 (IZ = 19 mm; MIC = 0.625 mg/mL), *B. cereus* ATCC 14579 (IZ= 24 mm; MIC = 1.25 mg/mL), *S. aureus* ATCC 25923 (IZ = 22 mm; MIC = 0.078 mg/mL), *S. epidermis* ATCC 12228 (IZ = 16 mm; MIC = 1.25 mg/mL), *E. faecalis* ATCC 29212 (IZ = 15 mm; MIC = 0.625 mg/mL), *Listeria monocytogenes* (IZ = 26 mm; MIC = 0.039 mg/mL), *Salmonella enterica* ATCC 43972 (IZ = 18 mm; MIC = 0.625 mg/mL), *E. coli* ATCC 25922 (IZ = 15 mm; MIC = 1.25 mg/mL), and *P. aeruginosa* ATCC 9027 (IZ = 14 mm; MIC = 2.5 mg/mL).

As essential oil is a mixture of large numbers of compounds belonging to various chemical classes; its antimicrobial activity may be related to the cumulative effects of major and/or minor components. Therefore, its antibacterial mechanism of action can be affected by several pathways on various cell organelles, such as the cell membrane and mitochondria, which may provoke cell death [38]. Its potentiality is highly correlated with the stability of its compounds, their volatility, their lipophilicity, and also their hydrophobicity in the wells. In our present study, although both EOL and EOP had the same major compound, limonene (60.6%, 86.8%), and the bacterial response was different, Gram-negative bacteria were found to be more resistant than Gram-positive bacteria. This variability in results has also been seen in previous studies, which showed that positive bacteria were more affected by EOs [38].

Limonene, the dominant compound in both *C. limon* and *C. paradisi* EOs, is well known to exhibit antibacterial effects [39]. Previous works have highlighted that oxygenated monoterpene such as alcohol and phenols had stronger bacterial inhibition effects than hydrocarbons, which may explain our findings [40].

In this study, we have also shown the anticandidal activities of *C. limon* and *C. paradisi* EOs tested against *C. glabrata* ATCC 90030, *C. albicans* ATCC 90028, *C. parapsilosis* ATCC 22019, and *C. krusei* ATCC 6258 using microdilution assays. Our findings show that the tested citrus EOs were active at high concentrations (MICs and MFCs values) as compared to bacterial strains. Previous reports have indicated that *C. limon* EO inhibited *Candida* species and dermatophytes. For instance, *C. tropicalis* (IZ = 23.61 mm, MIC = 0.017 mg/mL, MFC = 0.011 mg/mL), *Rhizopus nigricans* (14.15 mm) and *Aspergillus flavus* (MIC = 0.058 mg/L, MBC = 0.023 mg/mL) were active against EOL [41]. Similarly, *C. limon* EO prevented the growth of *A. niger* CTM 10099 (IZ= 26 mm; MIC = 0.625 mg/mL), *A. flavus* (IZ = 26 mm; MIC = 0.312 mg/mL), *A. nidulans* (IZ = 20 mm; MIC = 0.625 mg/mL), *A. fumigatus* (IZ = 18 mm; MIC = 0.625 mg/mL), *Fusarium graminearum* ISPAVE 271 (IZ = 20 mm; MIC = 0.625 mg/mL), *F. oxysporum* (CTM10402) (IZ = 18 mm; MIC = 0.625 mg/mL), *F. culmorum* ISPAVE 21w (IZ = 21 mm; MIC = 0.312 mg/mL) and *Alternaria alternata* CTM 10230 (IZ = 17 mm; MIC = 1.25 mg/mL) [11].

Recently, it has been shown that *C. limon* EO obtained from flowers exhibited antifungal activity against *Fusarium* sp. (MIC = 0.625 mg/mL) and *Aspergillus* sp. (MIC = 1.25 mg/mL) [11]. At the concentration of 0.94%, EOL and EOP totally inhibited the growth of dermatophytes *A. flavus*, *A. niger*, *Penicillium chrysogenum* and *P. verrucosum* [41]. EOP also exhibited a wide inhibitory effect on some fungi cultures. For instance, *C. albicans* was effective against EOP at 5, 10 and 20 µg/mL, with inhibition percentages 51.9, 70.1 and 123%, respectively [36].

As the major component of both oils was limonene, this substance is probably responsible for the antifungal proprieties of the oils. *R*-(+)-limonene and *S*-(−)-limonene have been reported to inhibit, respectively, *C. albicans* ATCC 90028 (MIC = 0.31 mg/mL, MFC = 0.62 mg/mL; MIC = 0.31 mg/mL, MFC = 0.62 mg/mL), *C. glabrata* ATCC 90030 (MIC = 0.31 mg/mL, MFC = 1.25 mg/mL; MIC = 0.62 mg/mL, MFC = 1.25 mg/mL), *C. parapsilosis* ATCC 22019 (MIC = 0.31 mg/mL, MFC = 1.25 mg/mL; MIC = 0.31 mg/mL, MFC = 1.25 mg/mL) and *C. krusei* ATCC 6258 (MIC = 0.07 mg/mL, MFC = 0.62 mg/mL; MIC = 0.15 mg/mL, MFC = 0.31 mg/mL) [42]. Limonene has been documented to possess antifungal effects. It induces damage in ADN and in the membrane of fungi, and induces apoptosis [43]. This compound can inhibit *C. albicans* with an MIC value of about 300 µg/mL and an MFC value of about 400 µg/mL. Limonene reduced the secretion of proteinases (73%) and phospholipases (53%), as well as adhesion (91%) and biofilm formation (87%) in *C. albicans* [44]. These important antifungal capacities of both volatile oils may be related to their main component (Limonene) or to the other chemical compounds present, such as pinene and myrcene.

The anticoagulant potentialities of the volatile oils show that *C. paradisi* EO is more active than *C. limon* EO. The complex relation between inflammation and coagulation makes these processes interdependent, showing a substantial impact on each other. The coagulation system can be activated by inflammation reactions. In natural defense systems, coagulation pathways may amplify the inflammatory response and thus prevent catastrophic events [45]. Previous reports have shown that monoterpenes exhibit anticoagulant activities by affecting the hemostasis process. They can reduce collagen-induced platelet aggregation, induce antithrombotic reactions and block the P2Y12 platelet receptor [46,47]. Citrus caused the inactivation of thrombin and a decrease in the concentration of fibrinogen. The inhibition of platelet aggregation caused by citrus was also registered in rabbits [48]. Thus, the components may intervene in any anticoagulant process, such as via the inhibition of platelet activation, and/or modify the adhesiveness of collagen and fibrinogen. The oils may also induce the S-sulhydration of platelet proteins [49]. However, there are no clear mechanisms of the anticoagulant effects of these two varieties of essential oils.

## 4. Materials and Methods

### 4.1. Sample Preparation

In this study, *C. paradisi* and *C. lemon* essential oils were purchased from Bio-orient, Tunisia. *C. limon* and *C. paradisi* were collected on 11 April 2022 from Hammamet; they had grown in sandy soil, without fertilization and human intervention via irrigation. They were sampled at the mature period of 5 years, showing yellow and orange colors, respectively.

The fresh *C. limon* and *C. paradisi* were washed and then peeled separately. After drying the peels at room temperature, the white inner membranes were eliminated. A sugarcane juice presser, from Taiwan, was used to press almost three kilograms of each species. The obtained juice was centrifuged at 6000 rpm for 30 min. Then, excess water was removed using anhydrous sodium sulfate.

### 4.2. GC–EIMS Analysis

GC-EIMS analyses of each cultivar were carried out using a Varian CP-3800 gas chromatograph equipped with a DB-5 capillary column (30 m × 0.25 mm; coating thickness 0.25 µm) and a Varian Saturn 2000 ion trap mass detector. Analytical conditions: injector and transfer line temperatures 220 and 240 °C, respectively; oven temperature programmed from 60 to 240 °C at 3 °C/min; carrier gas helium at 1 mL/min. Essential oils were injected at 0.2 mL (10% hexane solution), with a split ratio of 1:30; SPME employed an injector temperature of 250 °C, with splitless injection. The identification of the components was based on comparisons of the retention times with those of standard samples, comparing their linear retention indices relative to the series of n-hydrocarbons, and using computer matching against commercial (NIST 2000 and Adams 2007) and home-made library mass spectra composed of pure ingredients and constituents of known oils, along with data on MS from the literature [50]. Volatile compounds were assembled into classes of monoterpene hydrocarbons, oxygenated monoterpenes, oxygenated sesquiterpenes, sesquiterpene hydrocarbons and other compounds. Three repeated injections were performed in order to verify the data obtained from SPME and GC-MS.

### 4.3. Antimicrobial Activity

#### 4.3.1. Microbial Strains

The essentials oils were tested against *Pseudomonas aeruginosa* imipenem-resistant and methicillin-resistant *S. aureus* (MRSA), *Bacillus cereus* ATCC 14579, *B. cereus* ATCC 11778, *E. faecium* CI234, *E. faecalis* ATCC 29212, *K. pneumoniae* ATCC 13883, *A. baumannii* ATCC 19606, *E. coli* ATCC 25922, *S. aureus* ATCC 25923, *C. krusei* ATCC 6258, *C. albicans* ATCC 90028, *C. glabrata* ATCC 90030, and *C. parapsilosis* ATCC 22019. All the above species were kindly provided by the Laboratory of Microbiology, Hospital of Fattouma Bourguiba, Monastir.

#### 4.3.2. Microdilution Method

This technique was used to estimate the minimum inhibitory concentration (MIC) and minimal bactericidal (MBC), as previously described [51,52].

The volatile oils were dissolved in dimethyl sulfoxide (DMSO, 10%) and physiological water. The tested oils were diluted to the highest concentration (50 mg/mL) and serial dilutions were prepared. A volume of 100 µL of brain–heart broth (BHI Broth, Sigma-Aldrich, Steinheim, Germany) was added into the 96-well plates with the bacteria. Then, 20 µL of the overnight culture was adjusted to 0.5 McFarland turbidity. The plates were incubated for 24 h at 37 °C [49].

The MIC represents the lowest concentration of essential oil that could prevent any growth visible to the eye after 24 h of incubation at 37 °C. In addition, the MBC presents the weakest concentration of essential oil that could prevent any growth visible to the eye. The MBC/MIC ratio allowed us to determine the bactericidal and bacteriostatic profiles of the tested essential oils. If this ratio was greater than 4, the essential oil had a bacteriostatic effect, and the effect was bactericidal when this was lessthan or equal to 4 [23,35].

### 4.4. Anticoagulant Activity

In order to determine the prothrombin time (PT) and activated partial thromboplastin time (aPTT), we used assay kits for the determination of PT, thrombin time (TT) and activated partial thromboplastin time (APTT)—SINNOWA Medical Science & Technology Co., Ltd. (Nanjing, Jiangsu, China). The activity was evaluated according to the protocol described by [53,54]. Human blood was collected from the Laboratory of Hematology of Fattouma Bourguiba Hospital, Monastir. Then, it was anticoagulated using 3.8% tri-sodium citrate (Sigma-Aldrich, Steinheim, Germany) in a polypropylene container. Immediately after, it was centrifuged for 15 min.

The plasma was separated, pooled, and stored at 4 °C until its use. In total, 50 μL of the essential oil was mixed with 100 μL of plasma and incubated for 5 min at 37 °C. A volume of 200 μL of PT assay reagent (rabbit brain extract and calcium chloride), pre-warmed to 37 °C for 15 min, was added. The clotting time was determined using a coagulometer (RT-2204C Medical expo). For aPTT tests, plasma (100 μL) was mixed with 50 μL of each sample and 100 of aPTT reagent, and thereafter incubated at 37 °C for 2 min. A volume of 50 μL of calcium chloride (0.25 mM) (Sigma-Aldrich, Steinheim, Germany) was added, and the clotting time was measured using a coagulometer. Normal saline was used as the negative control, and heparin (1 IU/mL) was used as the positive control. The tests were performed in triplicate.

### 4.5. Molecular Docking Study

Molecular docking studies were carried out using the Auto Dock 4.2 program package [49]. ACD (3D viewer) 2017.2.1 software (http://www.filefacts.com/acd3d-viewer-freeware-info, accessed on 15 May 2023). was used to perform the optimization of all the geometries of the compounds. We obtained “the crystal structure of cytidine deaminase from *K pneumoniae*” (PDB: 6K63) with grid box parameters of 30 (x-dimension) × 30 (y-dimension) × 30 (z-dimension) Å along with the x, y and z values of 38.321, −75.601 and −9.572, respectively, and “the crystal structure of the C (30) carotenoid dehydrosqualene synthase from *S. aureus* complexed with bisphosphonate BPH-652” (PDB: 2ZCQ) [55] with grid box parameters of 30 (x-dimension) × 30 (y-dimension) × 30 (z-dimension) Å and x, y and z values of 15.285, 54.913 and 41.662, respectively, for antibacterial activity assessment from the RSCB protein data bank. During the preparation of the receptor input file, water molecules were erased and the missing hydrogen and Gasteiger charges were added to the system. The corresponding ligand and protein files (PDBQT) were prepared using AutoDock Tools.

After this, the pre-calculation of the grid maps was performed using Auto Grid to save time during docking. The visualizations and analyses of interactions and images depicting the results of the in silico analysis were constructed using the software BIOVIA Discovery Studio Visualizer 2017R2 (https://www.3dsbiovia.com/products/collaborative-science/biovia-discovery-studio/, accessed on 22 October 2023).

### 4.6. ADME properties

The pharmacokinetic and drug-likeness properties of the predominant constituents of the volatile oils of two varieties of citrus were estimated using ADME (absorption, distribution, metabolism and excretion) descriptors via a SwissADME online server (http://www.swissadme.ch/, accessed on 22 October 2023).

## 5. Conclusions

The chemical compositions of *C. limon* EO and *C. paradisi* EO, as analyzed by gas chromatography coupled to mass spectrometry, reveal that *C. paradisi* EO possessed a higher concentration of limonene than *C. limon* EO. In fact, *β*-pinene was found at a high concentration (13.3%) in *C. limon* EO as compared to *C. paradisi* EO (0.2%). The in vitro effectiveness of these essential oils as natural antimicrobial and anticoagulant agents was tested. In silico docking simulations were performed to investigate the modes of action of the major constituents of cold-pressed volatile oils against some bacterial stains. The results suggest these oils could be used as natural antimicrobial and anticoagulant agents. Further studies will be needed to more deeply understand the compounds’ mechanisms of action on bacteria and *Candida* species.

## Figures and Tables

**Table 1:** Chemical compositions (%) of *C. limon* and *C. paradisi* volatile oils determined by SPME/MS.

Composition	RI	EOL	EOP
α-Thujene	933	0.4	
α-Pinene	941	2.1	0.5
Camphene	955	0.1	
Sabinene	977	2.2	0.2
β-Pinene	982	13.3	0.2
Myrcene	993	1.9	0.5
Octanal	1003		0.2
δ-3-Carene	1013		0.1
α-Terpinene	1020	0.2	
*p*-Cymene	1028	0.9	0.2
Limonene	1032	60.6	86.8
*(E)*-β-ocimene	1052	0.1	
γ-Terpinene	1063	11.0	
Terpinolene	1090	0.5	
Linalool	1101		0.4
*Cis-p*-menth-2-en-1-ol	1123		0.8
*Cis*-limonene oxide	1136		1.9
*Trans*-limonene oxide	1140		2.4
Citronellal	1155	0.1	
α-Terpineol	1191	0.2	
Decanal	1206		0.3
*Trans*-carveol	1219		1
*Cis*-carveol	1230		0.8
Neral	1241	1.2	
Carvone	1244		1.4
Geranial	1271	2.0	
Linalyl acetate	1259		0.8
Neryl acetate	1365	0.7	
Geranyl acetate	1383	0.6	0.2
Caryophyllene oxide	1582		0.1
Nootkatone	1809		0.2
*Cis*-α-bergamotene	1416	0.1	
β-Caryophyllene	1419	0.4	
*Trans*-α-bergamotene	1437	0.5	
β-Bisabolene	1508	0.8	
Monoterpene hydrocarbons (%)		93.3	88.4
Oxygenated monoterpenes (%)		4.8	9.7
Sesquiterpene hydrocarbons (%)		1.8	0.0
Oxygenated sesquiterpenes (%)		0.0	0.3
Non-terpene derivatives (%)		0.0	0.5
Total identified compound (%)		99.9	98.9

RI: Retention Index; EOL: *C. limon* essential oil; EOP: *C. paradisi* essential oil.

**Table 2:** Antibacterial activity of *C. limon* and *C. paradisi* essential oils determined by disc diffusion and microdilution assays.

Tested Microorganisms	*C. limon* Essential Oil			*C. paradisi* Essential Oil			Ciprofloxacin		
	MIC *	MBC **	MBC/MIC Ratio	MIC	MBC	MBC/MIC Ratio	MIC	MBC	MBC/MIC Ratio
*Pseudomonas aeruginosa* BLSE 2	2.5	2.5	1	0.768	0.768	1	0.125	>0.125	-
*Pseudomonas aeruginosa* BLSE13	2.5	2.5	1	0.768	0.768	1	0.125	>0.125	-
*Pseudomonas aeruginosa* IMP24	2.5	2.5	1	0.768	0.768	1	0.125	>0.125	-
*Pseudomonas aeruginosa* ATCC 27853	2.5	2.5	1	0.768	0.768	1	0.125	>0.125	-
*Escherichia coli* ATCC 25922	1.25	2.5	2	0.384	0.384	1	0.062	0.062	1
*Klebsiella pneumoniae* ATCC 13883	0.625	1.25	2	0.192	0.192	1	0.125	>0.125	-
*Acinetobacter baumannii* ATCC 19606	2.5	2.5	1	0.192	0.192	1	0.031	0.125	4
*Enterococcu faecalis* ATCC 29212	1.25	2.5	2	0.384	0.384	1	0.125	>0.125	-
*Enterococcus faecium* CI234	1.25	2.5	2	0.384	0.384	1	0.125	>0.125	-
*Staphylococcus aureus* ATCC 25923	0.625	1.25	2	0.384	0.768	2	0.031	0.125	4
*Staphylococcus aureus* MRSA-3	1.25	2.5	2	0.384	0.768	2	0.031	0.125	4
*Staphylococcus aureus* MRSA-126	1.25	2.5	2	0.384	0.768	2	0.031	0.125	4
*Bacilus cereus* ATCC 11778	1.25	2.5	2	0.768	1.536	2	0.002	0.062	31
*Bacilus subtilis* ATCC 14579	1.25	2.5	2	0.768	0.768	1	0.002	0.062	31

MIC *: minimal inhibitory concentration expressed in mg/mL; MBC **: minimal bactericidal concentration expressed in mg/mL.

**Table 3:** Anti-*Candida* spp. activity (mg/mL) of *C. limon* and *C. paradisi* essential oils using microdilution assays. MIC and MFC values expressed in mg/mL.

*Candida* Species	*C. limon* EO		MFC/MIC Ratio	*C. paradisi* EO		MFC/MIC Ratio	Amphotericin B
	MIC	MFC		MIC	MFC		MIC
*C. glabrata* ATCC 90030	6.25	12.5	2	1.2	2.4	2	0.5
*C. albicans* ATCC 90028	6.25	12.5	2	1.2	2.4	2	0.5
*C. parapsilosis* ATCC 22019	6.25	12.5	2	1.2	2.4	2	0.5
*C. krusei* ATCC 6258	12.5	12.5	1	1.2	2.4	2	0.5

**Table 4:** Anticoagulant activities of *C. limon and C. paradisi* essential oils.

Essential Oil/Control	PT(s)	aPTT(s)
*C. paradisi* EO	82.21 ± 2.65	180.00 ± 1.20
*C. limon* EO	80.00 ± 1.67	38.50 ± 2.05
Normal saline	15.00 ± 2.87	34.40 ± 1.54
Heparin	40.80 ± 4.71	120.50 ± 1.18

**Table 5:** Docking binding energies (kcal/mol) of promising antibacterial agents.

Compound	Free Binding Energy of Pdb: 6K63 (kcal/mol)	Free Binding Energy of Pdb: 2ZCQ (kcal/mol)
α-Pinene	−4.4	−5.7
Sabinene	−4.1	−6.2
β-Pinene	−4.1	−5.9
Limonene	−4.6	−6.7
γ-Terpinene	−4.3	−6.8
Geranial	−4.1	−6.9
Ciprofloxacin (Standard)	−4.2	−6.7

**Table 6:** The ligand- and receptor-binding interaction patterns.

Ligand	Docking Results of Pdb 6K63: Interacting Amino Acids (Types of Interactions)	Docking Results of Pdb 2ZCQ: Interacting Amino Acids (Types of Interactions)
α-Pinene	His182 (b,c); Phe184 (4b); Pro210 (2a)	Phe22 (4b); Tyr41 (b); Ala134 (2a); Val137 (a); Leu164 (a)
Sabinene	His182 (4b); Phe184 (b); His208 (2b); Pro210 (a)	Phe22 (2b); Phe26 (2b); Val137 (2a); Leu141 (2a); Leu164 (a)
β-Pinene	His182 (3b,c); Ala204 (a); His208 (b); Pro210 (2a)	Phe22 (4b); Ala134 (a); Val137 (a); Leu164 (a)
Limonene	His182 (3b); Phe184 (2b); Cys202 (a); Ala204 (a); His208 (b); Pro210 (2a)	Phe22 (2b); Phe26 (b); Tyr41 (2b); Cys44 (a); Val137 (2a); Leu164 (a)
γ-Terpinene	His182 (b,c); Phe184 (2b); Cys202 (a); Ala204 (a); Pro210 (2a); Lys265 (a)	Met15 (a); Phe22 (b); Phe26 (b); Val37 (a); Tyr41 (b); Ala134 (a); Val137 (2a); Leu141 (2a); Leu164 (a)
Geranial	His182 (b,c); Cys202 (a); Ala204 (a); His208 (2b); Pro210 (a)	Met15 (a); Phe22 (2b); Phe26 (2b); Val37 (2a); Ile40 (a); Tyr41 (2b); Val137 (a); Gly138 (d); Leu141 (2a); Ala157 (e); Leu164 (a)
Ciprofloxacin (Standard)	Thr174 (d); His182 (f); His208 (d,f,g)	Phe22 (2f); Phe26 (b); Val37 (a); Tyr41 (b); Val133 (g); Ala134 (b); Val137 (2c); Leu141 (a); Gly161 (d)

Na: a = alkyl; N = number of alkyl interactions; Nb: b = Pi–alkyl; N = number of Pi–alkyl interactions; Nc: c = Pi–Sigma; N = number of Pi–Sigma interactions; Nd: d = carbon–hydrogen bond; N = number of carbon–hydrogen bonds; Ne: e = conventional hydrogen bond; N = number of conventional hydrogen bonds; Nf: f = Pi–Pi; N = number of Pi–Pi interactions; Ng: g = halogen; N = number of halogen interactions.

**Table 7:** Physicochemical properties, pharmacokinetics, drug-likeness, and lipophilicity of four selected compounds, according to Swiss ADME online server.

Entry	α-Pinene	Sabinene	β-Pinene	Limonene	γ-Terpinene	Geranial
GI absorption	Low	Low	Low	Low	Low	High
BBB permeant	Yes	Yes	Yes	Yes	Yes	Yes
P–gp substrate	No	No	No	No	No	No
CYP1A2 inhibitor	No	No	No	No	No	No
CYP2C19 inhibitor	No	No	No	No	No	No
CYP2C9 inhibitor	Yes	No	Yes	Yes	No	No
CYP2D6 inhibitor	No	No	No	No	No	No
CYP3A4 inhibitor	No	No	No	No	No	No
Log Kp (cm/s) ^a^	−3.95	−4.94	−4.18	−3.89	−3.94	−4.71
Lipinski	Yes	Yes	Yes	Yes	Yes	Yes
TPSA (Å^2^)	00.00	00.00	00.00	00.00	00.00	20.23
Consensus Log *P*o/w	3.44	3.25	3.42	3.37	3.35	2.74
Bioavailability Score	0.55	0.55	0.55	0.55	0.55	0.55

a: skin permeation.

## Data Availability

All data are presented in the manuscript.

## Supplementary Materials

The following supporting information can be downloaded at: https://www.mdpi.com/article/10.3390/ph16121669/s1. SM1. Chromatograms obtained for both *C. limon* and *C. paradisi* obtained by using by SPME/MS technique.
