# Supplementary material for: In Vitro Screening of Antimicrobial and Anti-Coagulant Activities, ADME Profiling, and Molecular Docking Study of Citrus limon L. and Citrus paradisi L. Cold-Pressed Volatile Oils
Source: Pharmaceuticals (Basel). 2023 Nov 30;16(12):1669. doi: 10.3390/ph16121669 (PMC10748103; doi:10.3390/ph16121669)
Supplement: Supplementary file 1 [file pharmaceuticals-16-01669-s001.zip › pharmaceuticals-2704862-supplementary.pdf]

In vitro screening of antimicrobial and anti-coagulant activities, ADME profiling, and molecular docking study of *Citrus limon* L. and *Citrus paradisi* L. cold-pressed volatile oils

Assia Hamdi[1], Mabrouk Horchani[2], Hichem Ben Jannet[2], Mejdi Snoussi[3,4], Emira Noumi[3,4], Nouha Bouali[3,4], Adel Kadri[5,6], Flavio Polito[7], Vincenzo De Feo[7*], Hayet Edziri[8]

[1]Laboratory of Chemical, Pharmaceutical and Pharmacological Development of Drugs, Faculty of Pharmacy, University of Monastir, Tunisia; hamdiessia@gmail.com (A.H.)

[2]Laboratory of Heterocyclic Chemistry, Natural Products and Reactivity, Team: Medicinal Chemistry and Natural Products (LR11ES39), Department of Chemistry, Faculty of Science of Monastir, University of Monastir, Avenue of Environment, 5019, Monastir, Tunisia; horchani.mabrouk@gmail.com (M.H.); hichem.bjannet@gmail.com (H.B.J.)

[3] Department of Biology, College of Science, University of Ha'il, P.O. Box 2440, Hail 2440, Saudi Arabia; nouhabouali82@gmail.com (N.B.); eb.noumi@uoh.edu.sa (E.N.); m.snoussi@uoh.edu.sa (M.S.)

[4] Medical and Diagnostic Research Centre, University of Ha'il, Hail 55473, Saudi Arabia

[5] College of Science and Arts in Baljurashi, Al Baha University, P.O. Box (1988), Al Baha, Saudi Arabia. lukadel@yahoo.fr (A.K.)

[6] Laboratory of Plant Biotechnology Applied to Crop Improvement, Faculty of Sciences of Sfax, University of Sfax, B.P. 11713000, Sfax, Tunisia

[7] Department of Pharmacy, University of Salerno, Via Giovanni Paolo II, 132, Fisciano, 84084 Salerno, Italy; fpolito@unisa.it (F.P.); defeo@unisa.it (V.D.F.)

[8] Laboratory of Transmissible Diseases and Biologically Active Substances, Faculty of Pharmacy, 5000, Monastir, Tunisia; jaziri_hayet@yahoo.fr (H.J.)

***Correspondence: defeo@unisa.it (V.D.F.)**

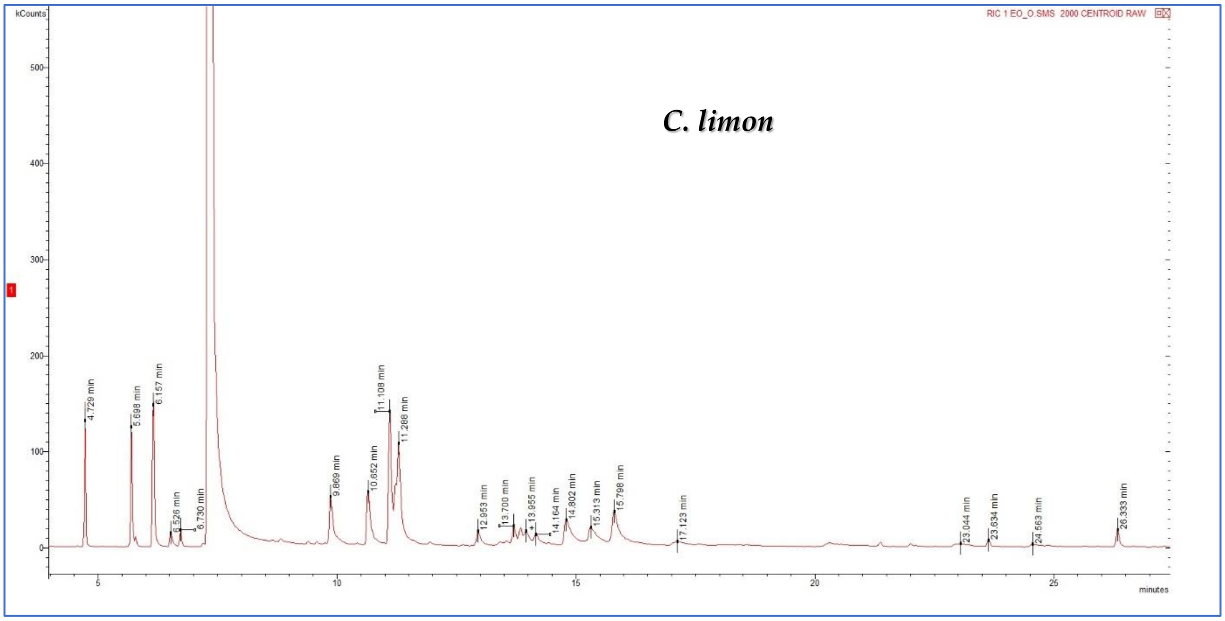

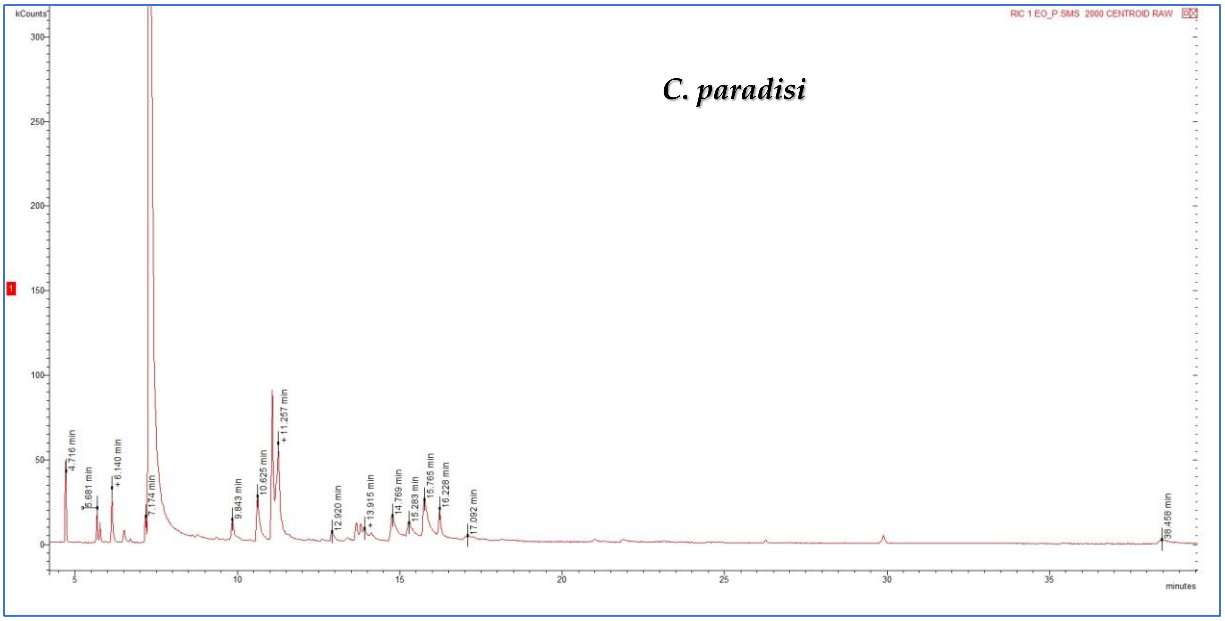

Supplementary material SM1. Chromatograms obtained for both C. limon and C. paradisi obtained by using by SPME/MS technique.
